# Ebolavirus Glycoprotein Fc Fusion Protein Protects Guinea Pigs against Lethal Challenge

**DOI:** 10.1371/journal.pone.0162446

**Published:** 2016-09-13

**Authors:** Krishnamurthy Konduru, Amy C. Shurtleff, Steven B. Bradfute, Siham Nakamura, Sina Bavari, Gerardo Kaplan

**Affiliations:** 1 Center for Biologics Evaluation and Research, Food and Drug Administration, Silver Spring, MD 20993, United States of America; 2 United States Army Medical Research Institute of Infectious Diseases, Fort Detrick, MD 21702, United States of America; Rockefeller University, UNITED STATES

## Abstract

Ebola virus (EBOV), a member of the *Filoviridae* that can cause severe hemorrhagic fever in humans and nonhuman primates, poses a significant threat to the public health. Currently, there are no licensed vaccines or therapeutics to prevent and treat EBOV infection. Several vaccines based on the EBOV glycoprotein (GP) are under development, including vectored, virus-like particles, and protein-based subunit vaccines. We previously demonstrated that a subunit vaccine containing the extracellular domain of the Ebola ebolavirus (EBOV) GP fused to the Fc fragment of human IgG1 (EBOVgp-Fc) protected mice against EBOV lethal challenge. Here, we show that the EBOVgp-Fc vaccine formulated with QS-21, alum, or polyinosinic-polycytidylic acid-poly-L-lysine carboxymethylcellulose (poly-ICLC) adjuvants induced strong humoral immune responses in guinea pigs. The vaccinated animals developed anti-GP total antibody titers of approximately 10^5^−10^6^ and neutralizing antibody titers of approximately 10^3^ as assessed by a BSL-2 neutralization assay based on vesicular stomatitis virus (VSV) pseudotypes. The poly-ICLC formulated EBOVgp-Fc vaccine protected all the guinea pigs against EBOV lethal challenge performed under BSL-4 conditions whereas the same vaccine formulated with QS-21 or alum only induced partial protection. Vaccination with a mucin-deleted EBOVgp-Fc construct formulated with QS-21 adjuvant did not have a significant effect in anti-GP antibody levels and protection against EBOV lethal challenge compared to the full-length GP construct. The bulk of the humoral response induced by the EBOVgp-Fc vaccine was directed against epitopes outside the EBOV mucin region. Our findings indicate that different adjuvants can eliciting varying levels of protection against lethal EBOV challenge in guinea pigs vaccinated with EBOVgp-Fc, and suggest that levels of total anti-GP antibodies elicit by protein-based GP subunit vaccines do not correlate with protection. Our data further support the development of Fc fusions of GP as a candidate vaccine for human use.

## Introduction

The *Filoviridae* is a family of zoonotic, filamentous, negative-strand RNA, enveloped viruses consisting of three genera: *Ebolavirus* and *Marburgvirus*, which can cause severe hemorrhagic fever in humans and nonhuman primates (NHPs) with high morbidity and mortality rates up to 90% [1–3], and *Cuevavirus*, which is pathogenic in bats and was recently discovered in Spain [4]. The rapid viral replication, immune suppression, multi-organ failure, vascular dysfunction, and progression to hemorrhagic fever are hallmarks of filovirus infection in primates [5, 6]. Filoviruses are BSL-4 pathogens classified as “Category A” bioterrorism agents, and currently there are no licensed therapeutics or vaccines to treat and prevent filovirus infection. *Marburgvirus* is antigenically stable and has a single species with two viruses, Marburg virus (MARV) and Ravn virus (RAVV), whereas *Ebolavirus* is more diverse and consists of five species, *Zaire*, *Sudan*, *Taï Forest*, *Reston*, and *Bundibugyo ebolavirus* each one with a single virus, Ebola virus (EBOV), Sudan virus (SUDV), Tai Forest virus (TAIFV), Reston virus (RSTV), and Bundibugyo virus (BDBV) [7]. RESTV is not pathogenic in humans but causes severe hemorrhagic fever in NHPs. In addition to primates, markers of natural ebolavirus infection have been detected in pigs, bats, dogs, duikers and perhaps some rodents (for a review, see [8]). It is likely that infected animals transmit EBOV to humans via contact with infected carcasses, exposure to aerosol or bat excreta within caves, or direct contact and aerosols from pigs [9–11].

The recent filovirus epidemic caused by a new isolate of EBOV, the Makona strain (EBOV/Mak), started in Guinea in 2013, spread to several countries in West Africa including Liberia and Sierra Leone, and claimed thousands of lives is declared the outbreak officially over in 2015 after a coordinated effort of local and international organizations [12, 13]. The magnitude and complexity of this EBOV epidemic underscores the urgent need to develop and approve efficacious vaccines and therapeutics against filoviruses.

The EBOV genome of approximately 19 kb that contains 7 genes: nucleoprotein (NP), VP35, VP40, glycoprotein (GP), VP30, VP24, and the polymerase (L) [14]. Transcriptional editing of the GP gene results in the expression of three partially overlapping proteins that share the first N-terminal 295 amino acids: sGP, GP, and ssGP ([15] and references therein). The GP is a type-I transmembrane glycoprotein that is cleaved into disulfide-linked GP1 and GP2 subunits. The mature GP forms homotrimers that are presented as spikes on the surface of infected cells and virions, and are responsible for receptor binding, viral entry, and immunity [16, 17]. Immunization with GP is sufficient to protect animals against ebolavirus lethal challenge in the mouse, guinea pig, and NHP models. Several GP-based vaccine candidates are currently under development such as virus-vectored vaccines [18, 19] and virus-like particles, which confer protection from lethal challenge in animal models including NHPs [20–29].

EBOV infection in humans elicits cellular and humoral immune responses (for a review, see [30]) that are early and vigorous in survivors. Fatal cases are associated with immune dysregulation and high viremia [31, 32]. Most vaccine candidates including vesicular stomatitis virus (VSV) and adenovirus vectored-vaccines induce moderate to high levels of anti-GP antibodies in NHPs (for a review, see [33]), which correlate with protection against lethal challenge in the rodent and NHP models [34–37]. Vaccine candidates including parainfluenza and Newcastle virus vectored-vaccines [38] and virus-like particles (VLPs) [21] induce significant levels of neutralizing anti-GP antibodies in NHPs. Because neutralizing antibodies are generated during ebolavirus infection in humans [39] and passive transfer of neutralizing monoclonal [40, 41] and polyclonal [42] antibodies protected NHPs against lethal ebolavirus challenge, vaccines that elicit neutralizing antibodies may add an additional layer of protection against ebolavirus infection. Adjuvants and immune modulators may also play a significant role in enhancing cellular, humoral, and neutralizing immune responses capable of protecting against ebolavirus infection.

We are currently developing a GP subunit vaccine based on the extracellular domain of GP fused to the Fc fragment of human IgG1 (EBOVgp-Fc). In mammalian cells, EBOVgp-Fc undergoes the complex posttranslational modifications of native GP such as furin cleavage, disulfide bonding of the GP1 and GP2 subunits, glycosylation, and trimer formation [43]. Mice immunized with EBOVgp-Fc developed robust humoral and cellular responses, including high levels of total and neutralizing anti-GP antibodies and GP-specific CD8+ T-cells that produced INF-γ. The EBOVgp-Fc vaccinated mice were protected against EBOV lethal challenge [43]. Here, we analyzed the EBOVgp-Fc vaccine in the EBOV guinea pig challenge model. Guinea pigs vaccinated with EBOVgp-Fc adjuvanted with QS-21, alum, or polyinosinic-polycytidylic acid-poly-L-lysine carboxymethylcellulose (poly-ICLC) developed strong humoral and neutralizing responses that conferred different degrees of protection against lethal challenge with EBOV performed under BSL-4 conditions. The poly-ICLC adjuvanted EBOVgp-Fc vaccine protected 100% of the guinea pigs against EBOV lethal challenge whereas EBOVgp-Fc formulated with QS-21 or alum only protected 60–70% of the animals. Our data show that a subunit vaccine containing purified GP fused to Fc is highly effective in protecting guinea pigs against EBOV lethal challenge and underscores the significant role of the adjuvant in achieving complete protection. These results further support the development of GP fused to Fc as a candidate filovirus vaccine.

## Materials and Methods

### Cells

Chinese hamster ovary (CHO) dihydrofolate reductase negative cells (ATCC, CRL-9096) stably transfected with cDNA of the EBOVgp-Fc fusion protein (CHO/EBOVgp-Fc) [43] were grown in Iscove’s Dulbecco’s modified Eagle medium supplemented with 10% fetal bovine serum (FBS). Monkey kidney Vero E6 cells (ATCC, CRL-1586) were grown in Eagle’s minimal essential medium (EMEM) supplemented with 10% FBS. Human embryonic kidney 293 (HEK293) cells (Invitrogen, Inc.) were maintained in Dulbecco’s modified Eagle medium (DMEM) supplemented with 10% FBS. Baby hamster kidney (BHK-21) cells (ATCC, CCL-10) were grown in DMEM supplemented with 5% FBS. BSR-T7 cells, which are BHK-21 cells that express bacteriophage T7 RNA-polymerase [44] (kindly provided by Dr. K. Conzelmann, Pettenkoffer Institute, Munich, Germany), were grown in DMEM supplemented with 5% FBS and 1 mg/ml geneticin (Invitrogen, Inc.).

### Plasmids

A plasmid containing the cDNA of the Mayinga EBOV GP (GenBank accession no. AF272001), pVR-1012-ZEBOV-GP [45], was kindly provided by G. Nabel, Vaccine Research Center, NIH. The GP cDNA in pVR-1012-ZEBOV-GP has eight adenosine residues at the RNA editing site needed to produce the full-length EBOV GP. Plasmids coding for the cDNA of the full-length VSV genome [pVSVFL(+)], envelope protein (VSVG) deleted virus genome (pVSVΔG), nucleoprotein (pBS-N), phosphoprotein (pBS-P), and L polymerase (pBS-L) [46] were kindly provided by J. Rose, Yale University. A plasmid containing the EBOV GP replacing the VSV-G protein in the VSV genome, pVSV-EBOVgp, was described previously and used to rescue replication-competent virus [43]. The following plasmids were constructed for this study using standard techniques of genetic engineering and synthetic oligonucleotides described in Table 1. All constructs were verified by automated nucleotide sequence analysis.

**Table 1:** Oligonucleotides used in plasmid constructions.

Name	Polarity	Use and characteristics	Oligonucleotide sequence (5’ to 3’)^a^
A	+	Amplification PCR fragment I coding for amino acids 1–311 of GP	**GCTAGC**AGTATGGGCGTTACAGGAATATTGCAGTTA
B	-	Amplification PCR fragment I coding for amino acids 1–311 of GP	TACAACTGTGAAAGACAACTCTTCACT
C	+	Amplification PCR fragment II coding for amino acids 307–311 and 463–676 of GP	TCTTTCACAGTTGTAAACACTCATCACCAAGATACCGGA
D	-	Amplification PCR fragment II coding for amino acids 307–311 and 463–676 of GP	**GCTAGC**CTAAAAGACAAATTTGCATATACAGAA
D-noNheI	-	Amplification PCR fragment III coding for amino acids 1–311 of GP	CTAAAAGACAAATTTGCATATACAGAA
E	+	Amplification PCR fragment IV coding for the GFP cDNA. Oligonuceotide contains 15 nucleotides complementary to 5’end of primer D-noNheI, the VSV transcription stop/start signal sequence, and nucleotides from the N-terminus of GFP	TGCAAATTTGTCTTTGCTAGGTATGAAAAAAACTAACAGATATCACGCTCGAGAATTAATTAGTATGGTGAGCAAGGGCGAGGAGCTGTTC
F	-	Amplification PCR fragment IV coding for the GFP cDNA. Oligonucleotide contains nucleotides from the C-terminus of GFP followed by a stop codon.	**GCTAGC**CTAGTTATCTAGATCCGGTGGATC

^a^Nhe I restriction sites in bold and underlined.

### pVSV-EBOVgpΔmuc

To rescue replication-competent VSVG-deleted recombinant VSV (rVSV) containing a mucin-deleted EBOV GP, we deleted the mucin-like region of the EBOV GP cDNA between amino acids 312–462 using overlapping PCR (Table 1). Briefly, a PCR fragment I coding for amino acids 1–311 of GP was amplified using pVR-1012-ZEBOV-GP as template and synthetic oligonucleotides A and B. Similarly, a PCR fragment II coding for amino acids 307–311 followed by 463–676 of GP was amplified from pVR-1012-ZEBOV-GP using primers C and D. PCR fragments I and II were annealed, and an EBOVgpΔmuc fragment was amplified using primers A and D. The EBOVgpΔmuc fragment was cut with NheI enzyme at the 5’ and 3’ ends, and cloned into the unique NheI site in pVSVΔG. The correct orientation construct was termed pVSV-EBOVgpΔmuc.

### pEF-EBOVgpΔmuc-Fc

We construct a plasmid to express the extracellular domain of the mucin-deleted EBOV GP fused to the Fc fragment of human IgG1 in cell transfectants. To do so, we used the EBOVgpΔmuc fragment described above and same strategy to construct the plasmid coding for the EBOVgp-Fc fusion protein [43]. The resulting plasmid, which was termed pEF-EBOVgpΔmuc-Fc, coded for amino acids 1–311 and 463–637 of the EBOV GP followed by the FLAG tag peptide DYKDDDDK and the Fc fragment of IgG1.

### pVSV-EBOVgp-GFP

**Fig 1:**
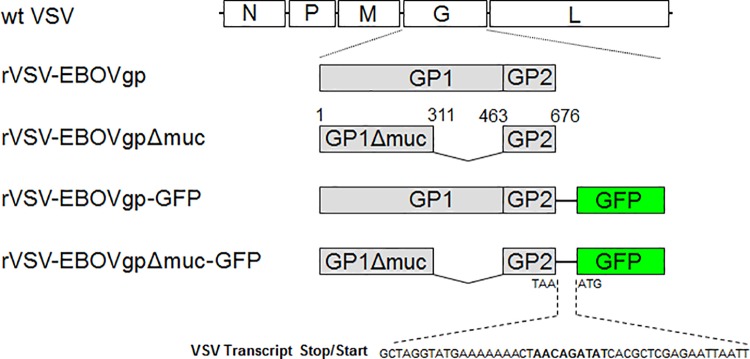
Schematic representation of recombinant vesicular stomatitis viruses (rVSV) containing the EBOV GP and green fluorescent protein (GFP). The VSV genome codes for the nucleoprotein (N), phosphoprotein (P), matrix (M), glycoprotein (G), and polymerase (L) genes. Viruses were rescued in BSR-T7 cells co-transfected with plasmids coding for N, P, and L, and plasmids containing the wild-type VSV (wt VSV) genome or constructs that replace the VSV-G gene for the full-length EBOV GP (rVSV-EBOVgp), mucin-like domain deleted EBOV GP (rVSV-EBOVgpΔmuc), EBOV GP followed by GFP (rVSV-EBOVgp-GFP, or mucin-deleted EBOV GP followed by GFP (rVSV-EBOVgp-GFP). The mucin-deleted EBOV GP contains amino acid residues 1–311 and 463–676 of EBOV GP. A VSV transcription stop/start signal (bold characters) was molecularly cloned between the GP and GFP genes to drive the expression of GFP.

To rescue rVSV containing the EBOV GP and GFP genes (Fig 1), we generated a cDNA fragment coding for both genes separated by a VSV transcription stop/start signal sequence [47] using overlapping PCR (Table 1). Briefly, EBOV GP cDNA was amplified from pVR-1012-ZEBOV-GP using synthetic oligonucleotides A and D-noNheI to produce PCR fragment III. Primers E and F were used to amplify PCR fragment IV containing the GFP cDNA (GenBank accession no. U57607.1). PCR fragments III and IV were annealed and amplified using primers A and F, the PCR product was digested at the 5’ and 3’ ends with NheI enzyme and cloned into the unique NheI in pVSVΔG. The correct orientation construct was termed pVSV-EBOVgp-GFP.

### pVSV-EBOVgpΔmuc-GFP

To rescue rVSV contained the mucin-deleted EBOV GP and GFP, we generated a mucin-deleted EBOV GP cDNA followed by the GFP cDNA (Fig 1) as described for the construction of pVSV-EBOVgp-Fc but using pVSV-EBOVgpΔmuc to amplify the mucin-deleted EBOV GP.

### Transfection and rescue of recombinant VSV (rVSV)

Replication competent rVSV in which the VSV-G protein was replaced by the EBOV GPΔmuc, EBOV GP plus GFP, or EBOV GPΔmuc plus GFP were generated using the VSV reverse genetics system [43, 48] and termed rVSV-EBOVgpΔmuc, rVSV-EBOVgp-GFP, and rVSV-EBOVgpΔmuc-GFP, respectively. Briefly, BSR-T7 cells were co-transfected with pVSV-EBOVgpΔmuc, pVSV-EBOVgp-GFP, or pVSV-EBOVgpΔmuc-GFP and pBS-N, pBS-P, and pBS-L using Lipofectamine 2000 (Invitrogen, Inc.) as a facilitator. After 48 h of incubation at 37°C, 50% confluent BHK-21 cell monolayers were infected with the supernatants of the transfected cells. All the rescued rVSV induced cytopathic effect in Vero E6 cells. Virus stocks were titrated in Vero E6 cells and stored at −80 C. Vero E6 monolayers infected with rVSV-EBOVgp-GFP or rVSV-EBOVgpΔmuc-GFP produced GFP, which was detected using an inverted fluorescence microscope.

### Production and purification of Fc fusion proteins

The FLAG-Fc control protein and the EBOVgp-Fc, a protein containing the extracellular domain of EBOV GP tagged with a DYKDDDDK (FLAG) peptide at the N-terminus and fused to the Fc and hinge fragment of human IgG1 were produced in CHO cell stable transfectants as described [43]. A similar construct containing the EBOV mucin-deleted construct EBOVgpΔmuc-Fc was produced in HEK293 cells transiently transfected with pEF-EBOVgpΔmuc-Fc using polyethylenimine PEI-Max MW 40,000 (Polysciences, Inc.) as a facilitator. To do so, transiently transfected HEK293 cells were grown overnight in DMEM containing 10% FBS. Monolayers were washed with DMEM, and cells were grown in serum-free OptiMEM medium (Invitrogen, Inc.). Supernatants of the HEK293 transfected cells were harvested 2–3 times at 24 h intervals. EBOVgp-Fc, EBOVgpΔmuc-Fc, and FLAG-Fc were secreted to the cell culture supernatant in serum-free medium and purified by affinity chromatography in protein A-agarose columns [43].

### Ethics Statement

Research was conducted in compliance with the Animal Welfare Act and other federal statutes and regulations following the principles stated in the Guide for the Care and Use of Laboratory Animals, 8^th^ Edition, National Research Council, 2011. The animal facility is fully accredited by the Association for Assessment and Accreditation of Laboratory Animal Care International. The USAMRIID Institutional Animal Care and Use Committee (IACUC) approved the animal protocol.

### Animal studies

Male and female guinea pigs approximately 350–450 g of body weight were used in the vaccination studies. Hartley strain Guinea pigs obtained from Charles River Labs were immunized using vaccine formulated with QS-21 (provided by a cooperative research agreement with Antigenics, Inc., now known as Agenus, Inc., Lexington, MA) as an adjuvant. Strain 13 guinea pigs obtained from an inbred colony maintained at the United States Army Medical Research Institute of Infectious Diseases (USAMRIID) were immunized using vaccine formulated with alum (Super-Alum; Novartis, Basel, Switzerland) or poly-ICLC (Hiltonol; Oncovir, Washington, DC) as adjuvants.

**Fig 2:**
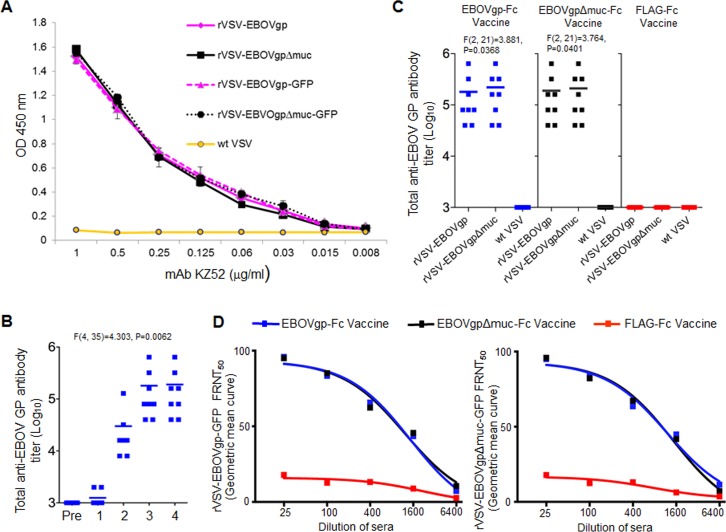
Anti-GP humoral response in strain 13 guinea pigs vaccinated with EBOVgp-Fc or EBOVgpΔmuc-Fc adjuvanted with QS-21. (A) Validation of the particle-to-TCID50 ratio and amount of GP incorporated in the virions used in the ELISA. Similar titers of rVSV-EBOVgp, rVSV-EBOVgpΔmuc, rVSV-EBOVgp-GFP, rVSV-EBOVgpΔmuc-GFP, and negative control wt VSV were used to coat the 96-well ELISA plate. Human anti-EBOV GP mAb KZ52 was titrated on the plates, and bound antibodies were stained with HRP-labeled anti-human IgG antibodies. Data are mean absorbance of duplicate wells, and bars represent standard errors of mean. (B) Kinetics of anti-GP antibody production in guinea pigs (n = 8) vaccinated with EBOVgp-Fc. Serum samples were collected before the primary immunization (Pre), the 2^nd^ vaccination at 21 days (1), the 3^rd^ vaccination at 42 days (2), the 4^th^ vaccination at 63 days (3), and at 77 days (4). Total anti-GP antibody titers were determined by an endpoint dilution virus particle ELISA in 96-well plates coated with rVSV-EBOVgp. (C) Total anti-GP antibodies in sera from guinea pigs vaccinated with EBOVgp-Fc (n = 8, blue squares), EBOVgpΔmuc-Fc (n = 8, gray squares), or control FLAG-Fc (n = 7, red squares). Sera samples were collected at day 63 prior to the final (third) boost. Total anti-EBOV GP antibodies in each animal were determined by an endpoint dilution virus particle ELISA. The mean for each group of vaccinated guinea pigs is shown as a line. Sera from the vaccinated guinea pigs were titrated by duplicates on 96-well plates coated with rVSV-EBOVgp, rVSV-EBOVgpΔmuc, and control wt VSV particles. (D) Analysis of anti-GP neutralizing antibodies by a BSL-2 fluorescence reduction neutralization test (FRNT). rVSV-EBOVgp-GFP (left panel) or rVSV-EBOVgpΔmuc-GFP (right panel) recombinant viruses were incubated with four-fold serial dilutions of sera from the vaccinated guinea pigs. Vero E6 cell monolayers were infected with these neutralization reactions, and the number of GFP fluorescent cells was assessed by flow cytometry 12 h after infection. The percent reduction in fluorescent cells compared to pre-immune serum treated virus was calculated as the % FRNT. The values are plotted in Geometric mean curve to measure FRNT50 titer. Differences in mean antibody titers were determined by ANOVA F-test between time points (B) and groups (C).

Guinea pigs were vaccinated on day 0, 21, 42, and 63 intramuscularly (i.m.). For the animals immunized using QS21 as an adjuvant, we used 100 μg/dose of EBOVgp-Fc, EBOVgpΔmuc-Fc, or control FLAG-Fc and 25 μg/dose of QS-21. For the animals immunized using alum or poly-ICLC as adjuvants, we used 50 μg/dose of EBOVgp-Fc, or control FLAG-Fc and 500 μg/dose of alum or poly-ICLC. The same number of animals was included in all vaccinated groups (8 guinea pigs/group). Because during the course of vaccination we determined that some animals were pregnant or died due to unrelated causes, these guinea pigs were not included in the challenge studies. Blood samples were collected from the guinea pigs immediately before each vaccination. For the anti-GP pilot study (Fig 2B), blood samples were also collected 14 days after the 4^th^ vaccination at day 91. The animals were challenged 14 days after the third boost at day 91 by intraperitoneal inoculation of 1,000 plaque-forming units (pfu) EBOV Mayinga adapted to guinea pigs (EBOV/May-GPA) diluted in PBS [49]. Guinea pigs were weighed and monitored for clinical signs as indicators of morbidity for approximately 24–26 days after challenge. All EBOV-infected guinea pigs were handled under maximum containment in a biosafety level-4 (BSL-4) laboratory at the USAMRIID, Frederick, MD. The dose and vaccination regimen was based on our previous work in mice [43]. Animals were monitored at least once a day and their status evaluated according to an Intervention Scoresheet approved by USAMRIID IACUC. Monitoring increased to three times in animals that scored three or four. Animals were euthanized by CO2 inhalation followed by confirmatory cervical dislocation. Analgesics and anesthetics were not used in this study and animals were euthanized for humane purposes if they reached a score of five or more, which would be indicated if the animals exhibited ruffled fur, weakness, unresponsiveness, and/or difficulty walking. Surviving animals were euthanized 26 days after challenge.

### Analysis of humoral Immune responses

The levels of EBOV GP-specific immunoglobulin G (IgG) antibodies in vaccinated guinea pigs were determined by a virus particle endpoint dilution ELISA [43] in 96-well plates coated with VSV pseudotypes. Briefly, 96-well plates were coated with 10^5^ TCID_50_ of rVSV-EBOVgp or rVSV-EBOVgpΔmuc produced in Vero E6 cells using serum-free OptiMEM medium. Plates coated with wt VSV were used as the specificity control. After blocked with PBS containing 5% bovine serum albumin (BSA), two-fold dilutions of the guinea pig sera were titrated in duplicates on the virus coated plates. Plates were incubated at 37°C for 1 h, washed and stained with horseradish peroxidase conjugated goat anti-guinea pig IgG (Jackson ImmunoResearch Laboratories, Inc) and 3,3',5,5'—tetramethylbenzidine SureBlue TMB substrate (KPL, Inc.). The colorimetric reaction was stopped with 1% sulfuric acid and absorbance was read in an ELISA plate reader at 450 nm. Antibody titers were defined as the highest dilution at which the mean absorbance of the sample was at least two-fold greater than the mean absorbance of the same sera dilution in control wells coated with wt VSV.

Anti-EBOV GP neutralizing antibodies were analyzed by a Fluorescence Reduction Neutralization Test (FRNT). To do so, 2,000 pfu of rVSV-EBOVgp-GFP or rVSV-EBOVgpΔmuc-GFP were treated with serum dilutions from the vaccinated animals in the presence of 5% guinea pig serum complement. After incubation at 37°C for 1 h, Vero E6 cell confluent monolayers in 6-well plates were inoculated in duplicates with each neutralization reaction. After adsorption for 1 h at 37°C, the inoculum was removed, cells were washed, DMEM containing 10% FBS was added, and plates were incubated for approximately 12 h at 37°C. Cells were detached by treatment with 1 ml of 0.5 mM EDTA in PBS, washed two times with PBS 2% FBS, and fixed with 2% paraformaldehyde. One million cells from each well were analyzed by flow cytometry for GFP fluorescence using a FACSCanto II cytometer (BD Biosciences). The percent neutralization (% FRNT) of each serum sample was calculated by comparing the number of GFP positive cells in monolayers infected with virus samples treated with pre-immune versus vaccinated serum using the formula 100-(vaccinated/pre-immune) x100. The serum dilution that reduced 50% of the fluorescent cells was defined as FRNT_50_. It should be pointed out that there is a high degree of correlation between the FRNT_50_ assay using rVSV-EBOVgp-GFP under BSL-2 conditions and the plaque reduction neutralization test (PRNT) using wild type EBOV/May (Konduru et al., submitted). To assure comparability between tested groups, we included internal controls using antibodies of known titers, which reacted similarly in assays from the different groups.

### Statistical analysis

Virus titers were calculated using the ID50 program developed by John L. Spouge (National Center for Biotechnology Information, NIH). Statistical significance between two groups was determined by unpaired t-test, and also verified by an F-test to compare variances, using the Prism version 6 program (GraphPad Software, Inc.). For more than two groups, the data was analyzed by ANOVA to calculate F value with the degrees of freedom and p-value (GraphPad software, Inc.). For multiple adjuvant groups, data was analyzed by ANOVA using the InStat program (GraphPad software, Inc.), and comparison of two treatments was performed using the Bonferroni test. All analysis of IgG responses were carried out on the log-scale. The FRNT50 was obtained using a 4-parameter logistic regression. Kaplan-Meier survival analysis was performed with the Prism version 6 program and evaluated using the Mantel-Cox test. Differences are considered non-significant (NS) when p≥0.05, significant (*) when p<0.05, and highly significant (**) when p<0.01.

## Results

### QS-21 adjuvanted EBOVgp-Fc and EBOVgpΔmuc-Fc vaccines induce strong humoral responses in guinea pigs

We previously showed that EBOVgp-Fc elicited humoral and cellular immune responses that protected mice against lethal challenge with EBOV [43]. Here, we analyzed the immunogenicity of EBOVgp-Fc in guinea pigs. We also studied the contribution of the mucin region of GP in protective responses by immunizing animals with the mucin-deleted GP construct EBOVgpΔmuc-Fc. Groups of 7–8 strain 13 guinea pigs were vaccinated at days 0, 21, 42, and 63 with 100 μg of EBOVgp-Fc, EBOVgpΔmuc-Fc, or control FLAG-Fc using QS-21 as adjuvant. Guinea pigs were bled before each boost, and total anti-GP antibodies were measured by a virus particle ELISA in plates coated with rVSV-EBOVgp, rVSV-EBOVgpΔmuc, or control wt VSV. It should be pointed out that we used a saturating amount of virus to coat the ELISA plates and that higher concentration of virus did not increase the signal. To control for the particle-to-TCID_50_ ratio of the different viruses and the amount of GP incorporated into the virus particles, we performed an ELISA using monoclonal antibody KZ52 (Fig 2A), which showed that similar amounts of GP were present in the wells irrespective of the recombinant VSV construct used to coat the wells. Our preliminary (Fig 2B) and historical [50] data indicated that anti-filovirus GP antibody titers reach a peak before the 4th vaccination at day 63, and that there are no significant differences in anti-GP antibody titers between day 63 and day 77 before challenge. The rationale for the 4^th^ vaccination is to increase T-cell immunity. To minimize stress of the animals, we did not bleed the guinea pigs at day 77 before challenge, so we analyzed anti-GP antibody titers in the 3^rd^ bleed obtained at day 63 before the last vaccination. The animals vaccinated with EBOVgp-Fc or EBOVgpΔmuc-Fc developed anti-GP antibody titers ranging from 1:40,000 to 1:640,000 whereas animals vaccinated with control FLAG-Fc did not develop anti-GP antibodies (Fig 2C). Similar levels of total anti-GP antibodies were produced in guinea pigs vaccinated with EBOVgp-Fc or EBOVgpΔmuc-Fc. Interestingly, sera from the EBOVgp-Fc or EBOVgpΔmuc-Fc vaccinated guinea pigs reacted similarly against rVSV-EBOVgp and rVSV-EBOVgpΔmuc. These results indicate that the bulk of the humoral response in the vaccinated guinea pigs was directed against non-mucin epitopes in GP.

We analyzed the anti-GP neutralizing antibodies in the sera of vaccinated guinea pigs by determining the effect of the sera in reducing the number of GFP fluorescent Vero E6 cells infected with rVSV-EBOVgp-GFP or rVSV-EBOVgpΔmuc-GFP compared to pre-immune sera as % FRNT. These replication-competent recombinant VSV particles contain the EBOV GP or GPΔmuc replacing the VSV-G envelope on the viral surface and the GFP gene cloned into the VSV genome (Fig 1). Therefore, neutralizing anti-GP antibodies that bind to rVSV-EBOVgp-GFP and rVSV-EBOVgpΔmuc-GFP prevent infection and the expression of GFP (Fig 2D). Antibodies from guinea pigs vaccinated with EBOVgp-Fc or EBOVgpΔmuc-Fc neutralized rVSV-EBOVgp-GFP (left panel) and rVSV-EBOVgpΔmuc-GFP (right panel) in a similar dose-dependent manner. Guinea pigs vaccinated with EBOVgp-Fc and EBOVgpΔmuc-Fc had FRNT50 titers 1442 and 1202 respectively against rVSV-EBOVgp-GFP, and titers 1187 and 1424 respectively against rVSV-EBOVgpΔmuc-GFP. Sera from guinea pigs vaccinated with FLAG-Fc resulted in background levels of neutralization of approximately 20% and had no FRNT50 titers. Taken together, these data indicated that EBOVgp-Fc and EBOVgpΔmuc-Fc vaccines elicited strong humoral responses resulting in high levels of anti-GP total and neutralizing antibodies.

### QS-21adjuvanted EBOVgp-Fc or EBOVgpΔmuc-Fc vaccines partially protect guinea pigs against lethal challenge with EBOV/May-GPA

**Fig 3:**
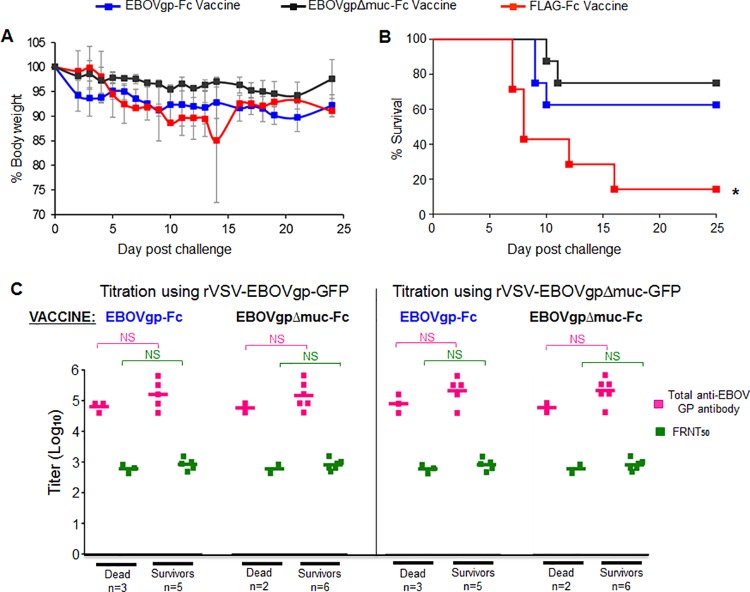
EBOV lethal challenge of strain 13 guinea pigs immunized with QS-21 adjuvanted EBOVgp-Fc or EBOVgpΔmuc-Fc vaccines. Groups of guinea pigs vaccinated with QS-21 adjuvanted EBOVgp-Fc (n = 8, blue squares), EBOVgpΔmuc-Fc (n = 8, gray squares), or FLAG-Fc (n = 7, red squares) were challenged with 1,000 pfu of EBOV/May-GPA two weeks after the third boost. (A) Weight loss after challenge with EBOV/May-GPA. Guinea pigs were weighed daily for 24 days. Data are group averages and bars indicate the standard errors of the mean. (B) Kaplan-Meier survival curves are plotted as percent survival for each vaccination group. Significant differences (*, P<0.05) between vaccinated (EBOVgp-Fc) and control (FLAG-Fc) animals as determined by the Mantel-Cox test. (C) Anti-GP total and neutralizing antibody titers of guinea pigs immunized with the EBOVgp-Fc or EBOVgpΔmuc-Fc vaccines and challenged with EBOV/May-GPA grouped according to the outcome in animals that died (Dead) or survived (Survivors) the challenge. Antibody titers were obtained using rVSV-EBOVgp-GFP (left panel) or rVSV-EBOVgpΔmuc-GFP (right panel) recombinant VSV constructs. The mean for each group is shown as a line, total anti-GP antibody titers (magenta squares), FRNT50 neutralizing antibody titers (green squares), and number of animals (n) per group. Significance in antibody titers were determined by unpaired t-test between groups; NS, not significant.

To analyze the protective effect of the EBOVgp-Fc and EBOVgpΔmuc-Fc vaccines formulated with the QS-21 adjuvant, the eight vaccinated guinea pigs per group were challenged two weeks after the third boost with 1,000 pfu of EBOV/May-GPA (Fig 3). Animals were observed for approximately 25 days post-challenge. The animals vaccinated with EBOVgp-Fc, EBOVgpΔmuc-Fc, and FLAG-Fc had a maximum weight loss of approximately 10%, 5%, and 15%, respectively (Fig 3A). Five (63%) and six (75%) of the guinea pigs vaccinated with EBOVgp-Fc and EBOVgpΔmuc-Fc, respectively, survived the EBOV/May-GPA lethal challenge (Fig 3B). In contrast, six out of the seven guinea pigs in the control group that received the control FLAG-Fc vaccine succumbed to the EBOV/May-GPA challenge. Analysis of the Kaplan-Meier survival curves revealed significant differences (P<0.05) between control (FLAG-Fc) and vaccinated (EBOVgp-Fc or EBOVgpΔmuc-Fc) animals.

We further analyzed the anti-GP total and neutralizing antibody levels stratifying the data according to the outcome of the challenge (Fig 3C). The BSL-2 virus particle ELISA and FRNT50 titers obtained using rVSV-EBOVgp-GFP (left panel) revealed that there are no significant differences in the levels of anti-GP total and neutralizing antibodies in survivors and dead guinea pigs immunized with the EBOVgp-Fc or EBOVgpΔmuc-Fc vaccines. Similar results were obtained when titrations were performed using rVSV-EBOVgpΔmuc-GFP (right panel).

### Alum and poly-ICLC adjuvanted EBOVgp-Fc vaccines elicit strong anti-GP humoral responses in guinea pigs

**Fig 4:**
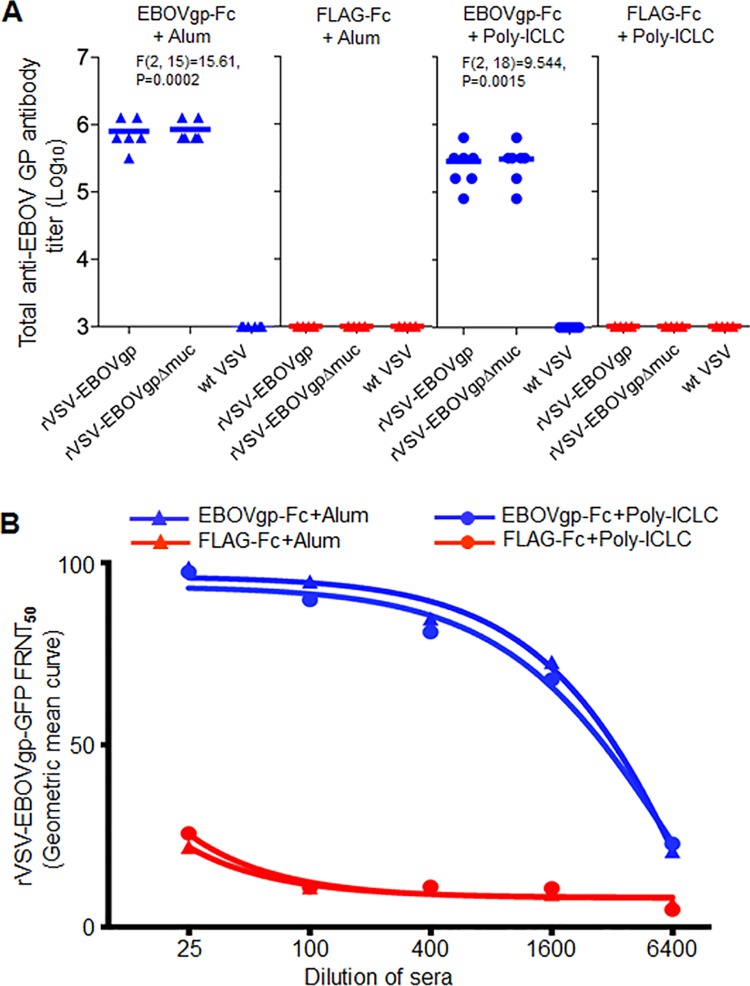
Anti-GP humoral response in Hartley guinea pigs immunized with alum or poly-ICLC adjuvanted EBOVgp-Fc vaccines. Guinea pigs were vaccinated with EBOVgp-Fc (n = 6 alum, n = 7 poly-ICLC) or control FLAG-Fc (n = 6) using alum (triangles) or poly-ICLC (circles) as adjuvants. Sera samples were collected at day 63 prior to the final (third) boost. (A) Analysis of total anti-EBOV GP antibodies by an endpoint dilution virus particle ELISA as in Fig 2C. (B) Analysis of anti-GP neutralizing antibodies by a BSL-2 FRNT. rVSV-EBOVgp-GFP was incubated with four-fold serial dilutions of sera from each guinea pig vaccinated with EBOVgp-Fc (blue) or FLAG-Fc (red) adjuvated with alum or poly-ICLC. Vero E6 cell monolayers were infected with the neutralization reactions, and the number of GFP fluorescent cells was assessed by flow cytometry and the FRNT50 was calculated by Geometric mean curves as in Fig 2C.

Because the QS-21 adjuvanted vaccines only induced partial protection in the challenged guinea pigs, we hypothesized that a different adjuvant could enhance the protective efficacy of our subunit vaccine. To test this hypothesis, we formulated our GP vaccine using alum or poly-ICLC adjuvants, which have different physicochemical characteristics and immune targets than QS-21. Since EBOVgp-Fc and EBOVgpΔmuc-Fc resulted in similar anti-GP antibody responses and protection levels (no significant differences), we focused vaccine development on EBOVgp-Fc because it contains the mucin region found in the natural forms of GP that may play a protective role in the NHP model and in humans. Hartley strain guinea pigs were vaccinated with 50 μg of EBOVgp-Fc or control FLAG-Fc adjuvanted with alum or poly-ICLC. Total anti-GP antibodies in the sera collected at day 63 just before the last boost were analyzed by the virus particle ELISA (Fig 4A). The EBOVgp-Fc vaccine adjuvanted with alum elicited total anti-GP antibody titers of 1:320,000 to 1:1,280,000. The poly-ICLC adjuvanted vaccine elicited titers that ranged from 1:80,000 to 1:640,000. The bulk of the antibody response was against GP epitopes outside the mucin region because similar ELISA titers were obtained in plates coated with the GP full-length (rVSV-EBOVgp) or mucin-deleted (rVSV-EBOVgpΔmuc) virus particles. As expected, the control FLAG-Fc adjuvanted vaccines did not elicit anti-GP specific antibodies.

We also analyzed anti-GP neutralizing antibodies in the sera from the vaccinated guinea pigs. To do so, rVSV-EBOVgp-GFP was treated with different dilutions of sera and neutralization was evaluated by FRNT50 (Fig 4B). The EBOVgp-Fc with alum and poly-ICLC adjuvanted vaccines had elicited FRNT50 titers 3136 and 3020 respectively. The control sera from FLAG-Fc vaccinated animals formulated with the same adjuvants showed background levels of neutralization of approximately 25% and had no FRNT50 antibody titers. These data indicated that EBOVgp-Fc elicited high humoral antibody responses in guinea pigs regardless of the adjuvant used in the formulation of the vaccine.

### Complete protection of guinea pigs immunized with the poly-ICLC adjuvanted EBOVgp-Fc vaccine

**Fig 5:**
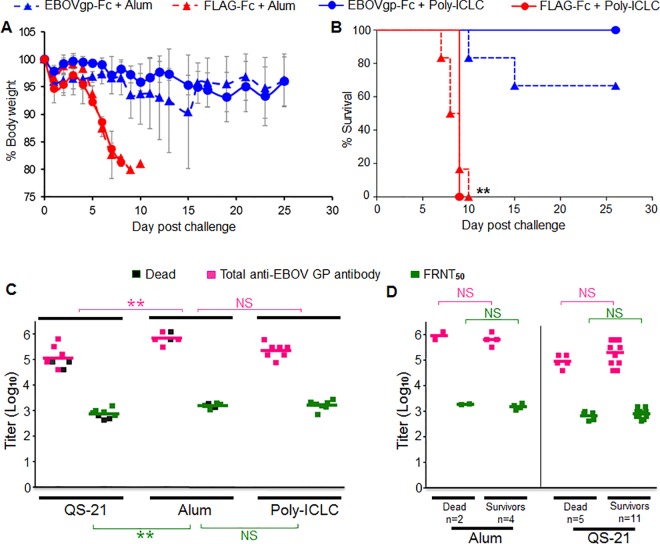
EBOV lethal challenge of guinea pigs immunized with EBOVgp-Fc vaccine using different adjuvants. Groups of guinea pigs vaccinated with alum (triangles) or poly-ICLC (circles) adjuvanted EBOVgp-Fc (n = 6 alum or n = 7 poly-ICLC, blue) or FLAG-Fc (n = 7, red) vaccines were challenged with 1,000 pfu of EBOV/May-GPA three weeks after the third boost. (A) Weight loss after challenge with EBOV. Guinea pigs were weighed for 25 days. Data are group averages and bars indicate the standard error of the mean. (B) Kaplan-Meier survival curves are plotted as percent survival for each vaccination group. Highly significant differences (**, P<0.01) between vaccinated (EBOVgp-Fc) and control (FLAG-Fc) animals as determined by the Mantel-Cox test. (C) Comparison of anti-GP humoral responses in guinea pigs vaccinated with EBOVgp-Fc using different adjuvants. The Hartley guinea pigs were used in the QS-21 experiments whereas strain 13 guinea pigs were used in the alum and poly-ICLC experiments. Titers of total anti-GP antibody (magenta) and neutralizing antibodies (FRNT_50_, green) elicited by the EBOVgp-Fc vaccine using QS-21 (data from Figs 2 and 3), alum (data from Figs 4 and 5B), or poly-ICLC (data from Figs 4 and 5B) are shown as squares for each vaccinated animal in a group. Guinea pigs that did not survive the lethal challenge are marked with gray squares. ANOVA and Bonferroni test were used to compare antibody levels between two adjuvant groups. Highly significant (**, p<0.01) and non-significant (NS) differences between two adjuvant groups are shown in the graph for anti-GP total (magenta) and neutralizing (green) antibodies. (D) Anti-GP total and neutralizing antibody titers of guinea pigs immunized with the EBOVgp-Fc (n = 6) vaccine adjuvanted with alum (left panel) or the EBOVgp-Fc (n = 8) or EBOVgpΔmuc-Fc (n = 8) vaccines adjuvanted with QS-21 and challenged with EBOV/May-GPA. Animals were grouped according to the outcome in animals that died (Dead) or survived (Survivors) the challenge. The animals immunized with the QS21-adjuvanted EBOVgp-Fc or EBOVgpΔmuc-Fc vaccines were combined into the Dead (n = 5) and Survivors (n = 11) groups. Antibody titers were obtained using the rVSV-EBOVgp-GFP recombinant VSV constructs. The mean for each group is shown as a line, total anti-GP antibody titers (magenta squares), FRNT50 neutralizing antibody titers (green squares), and number of animals (n) per group. Significance in antibody titers were determined by unpaired t-test between groups; NS, not significant.

The guinea pigs vaccinated with alum or poly-ICLC adjuvanted antigens were challenged 2 weeks after the third boost with 1,000 pfu of EBOV/May-GPA. The weight loss in animals vaccinated with EBOVgp-Fc was approximately 10% compared to the approximately 20% in guinea pigs vaccinated with FLAG-Fc vaccine (Fig 5A). The weight loss was less pronounced in the animals that received the poly-ICLC adjuvanted EBOVgp-Fc. Whereas animals that received the alum adjuvanted EBOVgp-Fc had higher loss of weight, one animal reached 20% weight loss. The guinea pigs that received the EBOVgp-Fc vaccine and survived the challenge regained some of the lost weight between days 15–25 post challenge. Mostly the animals started to gain weight at the end stage of the experimental timeframe. Two animals in the poly-ICLC group had gained weight to 100.8% at day-21 and 101.2% at day-25. Similarly two animals in the alum group had gained weight to 100.7% at day-17 and 101.7% at day-25. All the guinea pigs that received the poly-ICLC adjuvanted EBOVgp-Fc vaccine survived the lethal challenge (7/7) compared to the 67% (4/6) of the animals that received the alum adjuvanted EBOVgp-Fc vaccine (Fig 5B). The animals that received the control FLAG-Fc vaccine (6 animals with alum adjuvant and 6 animals with poly-ICLC adjuvant) did not survive the EBOV/May-GPA challenge. Analysis of the Kaplan-Meier survival curves revealed highly significant differences (p<0.01) between control and vaccinated animals. The weight loss and survival rates indicate that the poly-ICLC adjuvanted EBOVgp-Fc vaccine is highly effective in guinea pigs protecting 100% of the challenged animals with minimal morbidity.

### Analysis of correlates of protection

To analyze correlates of immunity in our study, we performed ANOVA of the anti-GP antibodies elicited by the EBOVgp-Fc vaccine using the different adjuvants (Fig 5C). It should be pointed out that the QS-21 challenge experiments were done using Hartley guinea pigs whereas the alum and poly-ICLC experiments were done using strain 13 guinea pigs. This analysis revealed that the QS-21 adjuvant elicited significantly lower levels of total (magenta squares) and neutralizing (green squares) antibodies compared to alum (p<0.01) but both adjuvants induced similar protection levels of approximately 65%. The same analysis showed that the alum and poly-ICLC adjuvanted vaccines elicited similar levels of anti-GP total and neutralizing antibodies. However, the poly-ICLC adjuvanted vaccine induced 100% protection against EBOV/May-GPA lethal challenge. Because all animals vaccinated with EBOVgp-Fc adjuvanted with poly-ICLC survived the lethal challenge with EBOV/May-GPA, we could not compare antibody levels in animals that survived or died as we did for the QS21-adjuvanted vaccine (Fig 5C). Therefore we focused our analysis on the antibody levels in survivors and dead animals that were immunized with QS21- or alum-adjuvanted vaccines. In Fig 3C, we performed a stratified analysis of antibody levels in guinea pigs immunized with the QS21-adjuvanted EBOVgp-Fc or EBOVgpΔmuc-Fc vaccines but, because these two vaccines behaved similarly, we combined the survivors (n = 11) and dead (n = 5) animals of both vaccines to increase the number of animals in both groups. The stratified analysis of total and neutralizing anti-GP antibodies according to the outcome also showed no significant differences in antibody levels between survivors and dead animals (Fig 5D, right panel). A similar stratified analysis of the antibody levels in guinea pigs immunized with the alum-adjuvanted EBOVgp-Fc vaccine also showed no significant differences in antibody levels between survivors and dead animals (Fig 5D, left panel). Although non-significant in the within alum group, the small sample size may not sufficient for robust analysis. Taken together, these data suggested that the adjuvant plays an important role in eliciting complete protection and indicate that antibody levels do not correlate with protection in this guinea pig model using our protein-based subunit GP vaccine.

## Discussion

During vaccine development, EBOV candidate vaccines are sequentially tested in the mouse, guinea pig, and NHP lethal challenge models to determine whether they can progress to clinical trials. Several EBOV GP vaccine candidates are currently under development (for a review, see [51]). EBOV GP vectored vaccine candidates based on VSV, rabies, human and chimpanzee adenovirus, parainfluenza, VEEV, and vaccinia were efficacious in the three animal models and some of them are currently undergoing clinical trials. Several other EBOV candidate vaccines not based on viral vectors, including VLPs and EBOV GP DNA have also shown efficacy in the three lethal challenge animal models. Our EBOVgp-Fc vaccine differs from the vectored vaccines in that it does not depend on replication of a viral vector to induce anti-EBOV immune responses and may result in less severe adverse effects. Compared to other non-vectored based EBOV GP vaccines, the EBOVgp-Fc vaccine is a well-characterized glycoprotein that can be produced in large quantities, purified to homogeneity, and delivered by simple immunization procedures. We have previously shown that the EBOVgp-Fc vaccine is highly efficacious in the mouse lethal challenge model [43], and in this paper in the guinea pig lethal challenge model. We are currently in the process of testing our vaccine candidate in the NHP challenge model, which is the most relevant model to human disease. Interestingly, the EBOVgp-Fc vaccine induced very high levels of anti-EBOV GP total and neutralizing antibody titers ranging from approximately 10^4.5^ to 10^6^ and 10^2.5^ to 10^3.5^, respectively, which is comparable to the antibody titers induced by other vectored and non-vectored vaccine candidates. Therefore, it is likely that the EBOVgp-Fc will also induce high anti-GP titers in NHPs that are commensurate with protection against lethal EBOV challenge as seen with other vectored and non-vectored EBOV candidate vaccines.

Here, we analyzed the importance of the adjuvant in conferring complete protection against EBOV/May-GPA lethal challenge in guinea pigs immunized with a protein-based GP subunit vaccine. In this study, we used the extracellular domain of EBOV GP fused to human IgG1 Fc, which may confer stability and enhance immunogenicity of GP, but did not analyze the contribution of the Fc fragment using constructs containing only the extracellular domain of GP. The same Fc fragment was fused to all constructs in this study to allow comparison between adjuvants. We vaccinated animals with the Fc fragment alone formulated with the different adjuvants as control to rule out the possibility of nonspecific protection resulting from immune responses against Fc. In animals immunized with EBOVgp-Fc, the use of poly-ICLC as an adjuvant increased the protective effect of the vaccine conferring complete protection whereas formulation with QS-21 or alum resulted in approximately 65% protection. It should be pointed out that we used 100 μg/dose of EBOVgp-Fc vaccine in the QS-21 group and 50 μg/dose in the alum and poly-ICLC groups. Because the QS21-adjuvanted vaccine elicited lower antibody levels and protection than the alum- or poly-ICLC-adjuvanted vaccines, the dose of EBOVgp-Fc was not responsible for the low performance of the QS21-adjuvanted vaccine. However, the use of strain 13 guinea pigs in the QS-21 experiments compared to the Hartley guinea pigs used in the alum and poly-ICLC experiments may have influenced the level of the antibody response. Statistical analysis revealed that there are no significant differences between the poly-ICLC, QS-21, and alum survival curves, so our data only points to the tendency of poly-ICLC to induce complete protection, which confirmation will require further investigation. The physicochemical characteristics and immune targets of the adjuvants played a significant role in inducing complete protection. Because limitations in space and number of animals per experiment under BSL-4 conditions, we could not evaluate all the adjuvants at the same time, and we did not compare the immunogenicity of GP constructs in the absence of an adjuvant. We first evaluated the effect of QS-21 using the Fc fusion proteins containing the full-length and mucin-deleted extracellular domains of GP. Our data showed that these two constructs induced similar antibody responses and protection levels, so we focused our study on Fc fusion construct containing the full-length extracellular domain of GP and analyzed the effect of the alum and poly-ICLC adjuvants.

The adjuvants that we used in this study have very different characteristics. Poly-ICLC, a synthetic polyinosinic:polycytidylic acid (poly-IC) double-stranded RNA stabilized with poly-L-lysine to increase RNase-resistance, is recognized by the cytosolic RNA helicase MDA-5 and the endosomal TLR3 and activate the production of type I IFN that stimulates B, T, and dendritic cells [52]. QS-21, a saponin derived from the bark of the South American soap tree *Quillaja saponaria*, is an amphipathic glycoside that acts as a surfactant and binds to cholesterol in biological membranes resulting in pore formation [53]. QS-21 is a potent adjuvant that increases the immunogenicity of pathogen and cancer vaccines by allowing cell entry of antigens to antigen-presenting cells (APCs) and also functioning as an irritant (for a review, see [54]). Alum (aluminum salts), the most commonly used adjuvant in human vaccines, adsorbs antigen onto its surface by electrostatic forces. The mechanism of action of alum is unclear but the efficient uptake of the adsorbed particulate antigen by APCs is among the proposed functions of this adjuvant [55]. It is possible that the detergent properties of QS-21 and the highly charged surface of alum induced changes in critical epitopes of EBOVgp-Fc affecting the protective efficacy of this antigen.

Poly-ICLC, QS-21, and alum have different immune stimulatory effects. Poly-ICLC induces the production of type I IFN that activates innate and adaptive immunity mechanisms resulting in strong antibody and T-helper 1 (Th1) responses [56]. QS-21 increases antigen presentation and has a pleiotropic effect that enhances antibody production, cytopathic T lymphocytes (CTL), and Th1 and T-helper 2 (Th2) responses [54]. Alum induces Th2 cellular and strong humoral responses but does not induce CTL [57]. Because Th1 and antibody responses play a significant role in protection against ebolavirus infection [34], the Th2 response induced by the alum adjuvant may have skewed the Th1 protective immune responses and be responsible for the partial protection observed in the vaccinated guinea pigs. The partial protection induced by the QS-21 adjuvant cannot be explained by the T helper characteristics of the immune response since QS-21 induces strong Th1 responses. The complete protection induced by the poly-ICLC adjuvanted EBOVgp-Fc vaccine could be due to the activation of specific components of the cellular immune response and/or targeting of protective epitopes that are not stimulated by alum or QS-21 adjuvants. Our work using the same protein-based antigen (EBOVgp-Fc) formulated with different adjuvants (QS-21, alum, or poly-ICLC) provides an excellent experimental model to identify correlates of immunity. Further work is needed to fully analyze the immune responses in the partially (QS-21 and alum) and complete (poly-ICLC) protected animals to identify differences in the immune response that could be correlated with protection.

Total anti-GP IgG antibody levels elicited by VSV and adenovirus vectored EBOV GP vaccines correlated with protection against EBOV/May-GPA lethal challenge in guinea pigs and NHPs [37]. However, correlates of protection in non-vectored GP vaccines have not been explored in great detail. Our data showed that the QS-21 adjuvanted EBOVgp-Fc vaccine induced a lower level of anti-GP antibodies compared to alum, and that the use of these two adjuvants resulted in partial (63–67%) protection against lethal EBOV/May-GPA challenge. The alum and poly-ICLC adjuvanted EBOVgp-Fc vaccines induced similar anti-GP total and neutralizing antibody responses but only the poly-ICLC induced complete protection whereas the alum adjuvanted vaccine protected 67% of the guinea pigs (4/6 animals) from lethal challenge with EBOV/May-GPA. Interestingly, the two guinea pigs in the alum group that died had very high anti-GP antibody levels. Taking together, these results suggested that there is a lack of correlation between protection and levels of anti-GP antibodies since the alum adjuvanted EBOVgp-Fc vaccine, which induced higher total anti-GP antibodies, resulted in similar levels of protection compared to the QS-21 adjuvanted vaccine. The stratified analysis of the QS-21 and alum groups according to the outcome of the challenge showed no significant differences in the levels of total and neutralizing anti-GP antibodies in survivors versus dead animal, which clearly indicated that there is no correlation between antibody levels and survival in guinea pigs immunized with our EBOVgp-Fc vaccines. It should be pointed out that we did not analyze the quality of the antibody response, which may also contribute to the difference in the survival outcome. Analysis of the epitopes targeted by the poly-ICLC adjuvanted EBOVgp-Fc vaccine compared to QS-21 and alum would help to determine whether poly-ICLC induced responses against specific protective epitopes of GP. Further work analyzing the immune responses in the partially (QS-21 and alum) and completely (poly-ICLC) protected animal groups is needed to identify differences in the immune response that could be correlated with protection.

The GP structure [58] suggests that the mucin region could mask epitopes in non-mucin regions of GP. However, the guinea pigs immunized with the EBOVgp-Fc or EBOVgpΔmuc-Fc vaccines adjuvanted with QS-21 produced similar levels of antibodies against non-mucin epitopes as assessed by the virus particle ELISA (total anti-GP antibodies) and the FRNT (neutralizing antibodies). These results indicate that the bulk of the antibody response against EBOVgp-Fc or EBOVgpΔmuc-Fc was directed against non-mucin epitopes. The EBOVgp-Fc and EBOVgpΔmuc-Fc vaccines induced similar protection levels suggesting that in the guinea pig model the mucin region of the EBOVgp-Fc vaccine does not play a significant role in eliciting protective immune responses. The immunodominant effect of the non-mucin epitopes was independent of the adjuvant because the EBOVgp-Fc vaccine formulated with QS-21, alum, or poly-ICLC induced similar levels of total antibodies against GP constructs with or without the mucin. Further research will be required to establish whether the immunodominant effect of non-mucin GP epitopes is an intrinsic characteristic of our subunit vaccine, and whether this immunodominant effect is restricted to guinea pigs or is also observed in mice and NHPs.

In humans and NHPs, the human IgG1 Fc fragment present in EBOVgp-Fc could enhance the immunogenicity of the vaccine by interacting with Fcγ receptors on antigen presenting cells [59–61]. The Fc tag may also increase the half-life and immunogenicity of the GP Fc fusion proteins as did in other systems [59, 62, 63]. Several antigen-Fc fusion proteins have been licensed and are being developed as therapeutics for human immune disorders and cancer (for a review, see [64]). The technology required to produce large amounts of the IgG1 Fc fusion proteins in mammalian cells using serum-free culture systems is readily available. Consequently, the use of Fc fusion proteins for the development of filovirus vaccines offers many advantages in terms of the adjuvant effect, stability, antigen production, etc., and a clear regulatory pathway for licensing.

In summary, we demonstrated that the poly-ICLC adjuvanted EBOVgp-Fc subunit vaccine is highly immunogenic and completely protected guinea pigs against lethal EBOV/May-GPA challenge. In mice, we previously showed that EBOVgp-Fc elicited anti-GP total and neutralizing antibodies as well as T-cell immunity that protected animals against lethal challenge with mouse-adapted EBOV[43]. This guinea pig study in conjunction with our previous work in mice shows that the EBOVgp-Fc is efficacious in two rodent models and suggests that this subunit vaccine could also show efficacy in the NHP model, which more closely resembles the human disease. EBOVgp-Fc is simple to produce, easy to purify, stable, and does not present issues regarding viral-vector pre-existing immunity and safety. Further work in NHPs will be required to determine whether the EBOVgp-Fc could be developed as a candidate vaccine for human use.

## References

[1] Feldmann H, Jones S, Klenk HD, Schnittler HJ. Ebola virus: from discovery to vaccine. Nat Rev Immunol. 2003;3(8):677–85. Epub 2003/09/17. 10.1038/nri1154 . 12974482

[2] Towner JS, Sealy TK, Khristova ML, Albarino CG, Conlan S, Reeder SA, et al Newly discovered ebola virus associated with hemorrhagic fever outbreak in Uganda. PLoS pathogens. 2008;4(11):e1000212 Epub 2008/11/22. 10.1371/journal.ppat.1000212 19023410 PMC2581435

[3] Baize S, Pannetier D, Oestereich L, Rieger T, Koivogui L, Magassouba N, et al Emergence of Zaire Ebola Virus Disease in Guinea. New Engl J Med. 2014;371(15):1418–25. 10.1056/NEJMoa1404505 . 24738640

[4] Kuhn JH, Becker S, Ebihara H, Geisbert TW, Johnson KM, Kawaoka Y, et al Proposal for a revised taxonomy of the family Filoviridae: classification, names of taxa and viruses, and virus abbreviations. Archives of virology. 2010;155(12):2083–103. Epub 2010/11/04. 10.1007/s00705-010-0814-x 21046175 PMC3074192

[5] Hartman AL, Towner JS, Nichol ST. Ebola and marburg hemorrhagic fever. Clin Lab Med. 2010;30(1):161–77. Epub 2010/06/02. 10.1016/j.cll.2009.12.001 . 20513546

[6] Shurtleff AC, Warren TK, Bavari S. Nonhuman primates as models for the discovery and development of ebolavirus therapeutics. Expert opinion on drug discovery. 2011;6(3):233–50. Epub 2011/03/01. 10.1517/17460441.2011.554815 . 22647202

[7] Kuhn JH, Bao Y, Bavari S, Becker S, Bradfute S, Brister JR, et al Virus nomenclature below the species level: a standardized nomenclature for laboratory animal-adapted strains and variants of viruses assigned to the family Filoviridae. Arch Virol. 2013;158(6):1425–32. Epub 2013/01/30. 10.1007/s00705-012-1594-2 ; PubMed Central PMCID: PMCPmc3669655. 23358612 PMC3669655

[8] Olson SH, Reed P, Cameron KN, Ssebide BJ, Johnson CK, Morse SS, et al Dead or alive: animal sampling during Ebola hemorrhagic fever outbreaks in humans. Emerg Health Threats J. 2012;5 Epub 2012/05/05. 10.3402/ehtj.v5i0.9134 22558004 PMC3342678

[9] Barrette RW, Xu L, Rowland JM, McIntosh MT. Current perspectives on the phylogeny of Filoviridae. Infect Genet Evol. 2011;11(7):1514–9. Epub 2011/07/12. 10.1016/j.meegid.2011.06.017 . 21742058 PMC7106080

[10] Heeney JL. Ebola: Hidden reservoirs. Nature. 2015;527(7579):453–5. 10.1038/527453a . 26607539

[11] Osterholm MT, Moore KA, Kelley NS, Brosseau LM, Wong G, Murphy FA, et al Transmission of Ebola viruses: what we know and what we do not know. MBio. 2015;6(2):e00137 10.1128/mBio.00137-15 25698835 PMC4358015

[12] Cenciarelli O, Pietropaoli S, Malizia A, Carestia M, D'Amico F, Sassolini A, et al Ebola virus disease 2013–2014 outbreak in west Africa: an analysis of the epidemic spread and response. Int J Microbiol. 2015;2015:769121 Epub 2015/04/09. 10.1155/2015/769121 ; PubMed Central PMCID: PMCPmc4380098. 25852754 PMC4380098

[13] Spengler JR, Ervin ED, Towner JS, Rollin PE, Nichol ST. Perspectives on West Africa Ebola Virus Disease Outbreak, 2013–2016. Emerging infectious diseases. 2016;22(6). 10.3201/eid2206.160021 . 27070842 PMC4880067

[14] Volchkov VE, Volchkova VA, Dolnik O, Feldmann H, Klenk HD. Polymorphism of filovirus glycoproteins. Adv Virus Res. 2005;64:359–81. Epub 2005/09/06. 10.1016/S0065-3527(05)64011-0 . 16139600

[15] Mehedi M, Falzarano D, Seebach J, Hu X, Carpenter MS, Schnittler HJ, et al A new Ebola virus nonstructural glycoprotein expressed through RNA editing. Journal of virology. 2011;85(11):5406–14. Epub 2011/03/18. JVI.02190-10 [pii] 10.1128/JVI.02190-10 21411529 PMC3094950

[16] Feldmann H, Volchkov VE, Volchkova VA, Stroher U, Klenk HD. Biosynthesis and role of filoviral glycoproteins. The Journal of general virology. 2001;82(Pt 12):2839–48. Epub 2001/11/21. . 11714958 10.1099/0022-1317-82-12-2839

[17] Takada A, Robison C, Goto H, Sanchez A, Murti KG, Whitt MA, et al A system for functional analysis of Ebola virus glycoprotein. Proceedings of the National Academy of Sciences of the United States of America. 1997;94(26):14764–9. Epub 1998/02/07. 9405687 10.1073/pnas.94.26.14764 PMC25111

[18] De Santis O, Audran R, Pothin E, Warpelin-Decrausaz L, Vallotton L, Wuerzner G, et al Safety and immunogenicity of a chimpanzee adenovirus-vectored Ebola vaccine in healthy adults: a randomised, double-blind, placebo-controlled, dose-finding, phase 1/2a study. Lancet Infect Dis. 2016;16(3):311–20. 10.1016/S1473-3099(15)00486-7 . 26725450

[19] Agnandji ST, Huttner A, Zinser ME, Njuguna P, Dahlke C, Fernandes JF, et al Phase 1 Trials of rVSV Ebola Vaccine in Africa and Europe. The New England journal of medicine. 2016;374(17):1647–60. 10.1056/NEJMoa1502924 . 25830326 PMC5490784

[20] Warfield KL, Bosio CM, Welcher BC, Deal EM, Mohamadzadeh M, Schmaljohn A, et al Ebola virus-like particles protect from lethal Ebola virus infection. Proceedings of the National Academy of Sciences of the United States of America. 2003;100(26):15889–94. Epub 2003/12/16. 10.1073/pnas.2237038100 14673108 PMC307663

[21] Warfield KL, Swenson DL, Olinger GG, Kalina WV, Aman MJ, Bavari S. Ebola virus-like particle-based vaccine protects nonhuman primates against lethal Ebola virus challenge. The Journal of infectious diseases. 2007;196 Suppl 2:S430–7. Epub 2007/12/06. 10.1086/520583 . 17940980

[22] Sullivan NJ, Geisbert TW, Geisbert JB, Shedlock DJ, Xu L, Lamoreaux L, et al Immune protection of nonhuman primates against Ebola virus with single low-dose adenovirus vectors encoding modified GPs. PLoS Med. 2006;3(6):e177 Epub 2006/05/11. 10.1371/journal.pmed.0030177 16683867 PMC1459482

[23] Sullivan NJ, Geisbert TW, Geisbert JB, Xu L, Yang ZY, Roederer M, et al Accelerated vaccination for Ebola virus haemorrhagic fever in non-human primates. Nature. 2003;424(6949):681–4. Epub 2003/08/09. 10.1038/nature01876 . 12904795 PMC7095492

[24] Sullivan NJ, Sanchez A, Rollin PE, Yang ZY, Nabel GJ. Development of a preventive vaccine for Ebola virus infection in primates. Nature. 2000;408(6812):605–9. Epub 2000/12/16. 10.1038/35046108 . 11117750

[25] Bukreyev A, Rollin PE, Tate MK, Yang L, Zaki SR, Shieh WJ, et al Successful topical respiratory tract immunization of primates against Ebola virus. Journal of virology. 2007;81(12):6379–88. Epub 2007/04/13. 10.1128/JVI.00105-07 17428868 PMC1900097

[26] Hevey M, Negley D, Pushko P, Smith J, Schmaljohn A. Marburg virus vaccines based upon alphavirus replicons protect guinea pigs and nonhuman primates. Virology. 1998;251(1):28–37. Epub 1998/11/14. 10.1006/viro.1998.9367 . 9813200

[27] Jones SM, Feldmann H, Stroher U, Geisbert JB, Fernando L, Grolla A, et al Live attenuated recombinant vaccine protects nonhuman primates against Ebola and Marburg viruses. Nature medicine. 2005;11(7):786–90. Epub 2005/06/07. 10.1038/nm1258 . 15937495

[28] Feldmann H, Jones SM, Daddario-DiCaprio KM, Geisbert JB, Stroher U, Grolla A, et al Effective post-exposure treatment of Ebola infection. PLoS pathogens. 2007;3(1):e2 Epub 2007/01/24. 10.1371/journal.ppat.0030002 17238284 PMC1779298

[29] Halfmann P, Ebihara H, Marzi A, Hatta Y, Watanabe S, Suresh M, et al Replication-deficient ebolavirus as a vaccine candidate. Journal of virology. 2009;83(8):3810–5. Epub 2009/02/13. 10.1128/JVI.00074-09 19211761 PMC2663241

[30] Bradfute SB, Bavari S. Correlates of immunity to filovirus infection. Viruses. 2011;3(7):982–1000. Epub 2011/10/14. 10.3390/v3070982 viruses-03-00982 [pii]. 21994766 PMC3185794

[31] Baize S, Leroy EM, Georges-Courbot MC, Capron M, Lansoud-Soukate J, Debre P, et al Defective humoral responses and extensive intravascular apoptosis are associated with fatal outcome in Ebola virus-infected patients. Nature medicine. 1999;5(4):423–6. Epub 1999/04/15. 10.1038/7422 . 10202932

[32] Sanchez A, Lukwiya M, Bausch D, Mahanty S, Sanchez AJ, Wagoner KD, et al Analysis of human peripheral blood samples from fatal and nonfatal cases of Ebola (Sudan) hemorrhagic fever: cellular responses, virus load, and nitric oxide levels. Journal of virology. 2004;78(19):10370–7. Epub 2004/09/16. 10.1128/JVI.78.19.10370-10377.2004 15367603 PMC516433

[33] Bradfute SB, Dye JM Jr., Bavari S. Filovirus vaccines. Hum Vaccin. 2011;7(6):701–11. Epub 2011/04/27. 15398 [pii]. 21519188 10.4161/hv.7.6.15398 PMC3219077

[34] Sullivan NJ, Martin JE, Graham BS, Nabel GJ. Correlates of protective immunity for Ebola vaccines: implications for regulatory approval by the animal rule. Nat Rev Microbiol. 2009;7(5):393–400. Epub 2009/04/17. 10.1038/nrmicro2129 . 19369954 PMC7097244

[35] Marzi A, Engelmann F, Feldmann F, Haberthur K, Shupert WL, Brining D, et al Antibodies are necessary for rVSV/ZEBOV-GP-mediated protection against lethal Ebola virus challenge in nonhuman primates. Proc Natl Acad Sci U S A. 2013;110(5):1893–8. Epub 2013/01/16. 10.1073/pnas.1209591110 ; PubMed Central PMCID: PMCPmc3562844. 23319647 PMC3562844

[36] Qiu X, Fernando L, Alimonti JB, Melito PL, Feldmann F, Dick D, et al Mucosal immunization of cynomolgus macaques with the VSVDeltaG/ZEBOVGP vaccine stimulates strong ebola GP-specific immune responses. PLoS One. 2009;4(5):e5547 Epub 2009/05/15. 10.1371/journal.pone.0005547 19440245 PMC2678264

[37] Wong G, Richardson JS, Pillet S, Patel A, Qiu X, Alimonti J, et al Immune parameters correlate with protection against ebola virus infection in rodents and nonhuman primates. Sci Transl Med. 2012;4(158):158ra46 10.1126/scitranslmed.3004582 23115355 PMC3789651

[38] DiNapoli JM, Yang L, Samal SK, Murphy BR, Collins PL, Bukreyev A. Respiratory tract immunization of non-human primates with a Newcastle disease virus-vectored vaccine candidate against Ebola virus elicits a neutralizing antibody response. Vaccine. 2010;29(1):17–25. Epub 2010/11/03. S0264-410X(10)01493-3 [pii] 10.1016/j.vaccine.2010.10.024 . 21034822 PMC3428043

[39] Maruyama T, Rodriguez LL, Jahrling PB, Sanchez A, Khan AS, Nichol ST, et al Ebola virus can be effectively neutralized by antibody produced in natural human infection. Journal of virology. 1999;73(7):6024–30. Epub 1999/06/11. 10364354 10.1128/jvi.73.7.6024-6030.1999 PMC112663

[40] Qiu X, Audet J, Wong G, Pillet S, Bello A, Cabral T, et al Successful treatment of ebola virus-infected cynomolgus macaques with monoclonal antibodies. Science translational medicine. 2012;4(138):138ra81 Epub 2012/06/16. 10.1126/scitranslmed.3003876 . 22700957

[41] Olinger GG Jr., Pettitt J, Kim D, Working C, Bohorov O, Bratcher B, et al Delayed treatment of Ebola virus infection with plant-derived monoclonal antibodies provides protection in rhesus macaques. Proc Natl Acad Sci U S A. 2012 10.1073/pnas.1213709109 . 23071322 PMC3497800

[42] Dye JM, Herbert AS, Kuehne AI, Barth JF, Muhammad MA, Zak SE, et al Postexposure antibody prophylaxis protects nonhuman primates from filovirus disease. Proceedings of the National Academy of Sciences of the United States of America. 2012;109(13):5034–9. Epub 2012/03/14. 10.1073/pnas.1200409109 22411795 PMC3323977

[43] Konduru K, Bradfute SB, Jacques J, Manangeeswaran M, Nakamura S, Morshed S, et al Ebola virus glycoprotein Fc fusion protein confers protection against lethal challenge in vaccinated mice. Vaccine. 2011;29(16):2968–77. Epub 2011/02/19. 10.1016/j.vaccine.2011.01.113 21329775 PMC3070761

[44] Buchholz UJ, Finke S, Conzelmann KK. Generation of bovine respiratory syncytial virus (BRSV) from cDNA: BRSV NS2 is not essential for virus replication in tissue culture, and the human RSV leader region acts as a functional BRSV genome promoter. Journal of virology. 1999;73(1):251–9. Epub 1998/12/16. 9847328 10.1128/jvi.73.1.251-259.1999 PMC103829

[45] Yang ZY, Duckers HJ, Sullivan NJ, Sanchez A, Nabel EG, Nabel GJ. Identification of the Ebola virus glycoprotein as the main viral determinant of vascular cell cytotoxicity and injury. Nature medicine. 2000;6(8):886–9. Epub 2000/08/10. 10.1038/78645 . 10932225

[46] Lawson ND, Stillman EA, Whitt MA, Rose JK. Recombinant vesicular stomatitis viruses from DNA. Proceedings of the National Academy of Sciences of the United States of America. 1995;92(10):4477–81. Epub 1995/05/09. 7753828 10.1073/pnas.92.10.4477 PMC41967

[47] Schnell MJ, Buonocore L, Whitt MA, Rose JK. The minimal conserved transcription stop-start signal promotes stable expression of a foreign gene in vesicular stomatitis virus. Journal of virology. 1996;70(4):2318–23. Epub 1996/04/01. 8642658 10.1128/jvi.70.4.2318-2323.1996 PMC190073

[48] Garbutt M, Liebscher R, Wahl-Jensen V, Jones S, Moller P, Wagner R, et al Properties of replication-competent vesicular stomatitis virus vectors expressing glycoproteins of filoviruses and arenaviruses. Journal of virology. 2004;78(10):5458–65. Epub 2004/04/29. 15113924 10.1128/JVI.78.10.5458-5465.2004 PMC400370

[49] Connolly BM, Steele KE, Davis KJ, Geisbert TW, Kell WM, Jaax NK, et al Pathogenesis of experimental Ebola virus infection in guinea pigs. The Journal of infectious diseases. 1999;179 Suppl 1:S203–17. Epub 1999/02/13. 10.1086/514305 . 9988186

[50] Warfield KL, Swenson DL, Negley DL, Schmaljohn AL, Aman MJ, Bavari S. Marburg virus-like particles protect guinea pigs from lethal Marburg virus infection. Vaccine. 2004;22(25–26):3495–502. Epub 2004/08/17. 10.1016/j.vaccine.2004.01.063 . 15308377

[51] Marzi A, Feldmann H. Ebola virus vaccines: an overview of current approaches. Expert Rev Vaccines. 2014;13(4):521–31. Epub 2014/03/01. 10.1586/14760584.2014.885841 . 24575870 PMC4785864

[52] Caskey M, Lefebvre F, Filali-Mouhim A, Cameron MJ, Goulet JP, Haddad EK, et al Synthetic double-stranded RNA induces innate immune responses similar to a live viral vaccine in humans. J Exp Med. 2011;208(12):2357–66. 10.1084/jem.20111171 22065672 PMC3256967

[53] Bangham AD, Horne RW, Glauert AM, Dingle JT, Lucy JA. Action of saponin on biological cell membranes. Nature. 1962;196:952–5. . 13966357 10.1038/196952a0

[54] Ragupathi G, Gardner JR, Livingston PO, Gin DY. Natural and synthetic saponin adjuvant QS-21 for vaccines against cancer. Expert Rev Vaccines. 2011;10(4):463–70. 10.1586/erv.11.18 21506644 PMC3658151

[55] Gupta RK. Aluminum compounds as vaccine adjuvants. Adv Drug Deliv Rev. 1998;32(3):155–72. . 10837642 10.1016/s0169-409x(98)00008-8

[56] Stahl-Hennig C, Eisenblätter M, Jasny E, Rzehak T, Tenner-Racz K, Trumpfheller C, et al Synthetic double-stranded RNAs are adjuvants for the induction of T helper 1 and humoral immune responses to human papillomavirus in rhesus macaques. PLoS Pathog. 2009;5(4):e1000373 10.1371/journal.ppat.1000373 19360120 PMC2660151

[57] Brewer JM. (How) do aluminium adjuvants work? Immunol Lett. 2006;102(1):10–5. Epub 2005/09/29. 10.1016/j.imlet.2005.08.002 . 16188325

[58] Lee JE, Fusco ML, Hessell AJ, Oswald WB, Burton DR, Saphire EO. Structure of the Ebola virus glycoprotein bound to an antibody from a human survivor. Nature. 2008;454(7201):177–82. Epub 2008/07/11. 10.1038/nature07082 18615077 PMC2700032

[59] Zhang MY, Wang Y, Mankowski MK, Ptak RG, Dimitrov DS. Cross-reactive HIV-1-neutralizing activity of serum IgG from a rabbit immunized with gp41 fused to IgG1 Fc: possible role of the prolonged half-life of the immunogen. Vaccine. 2009;27(6):857–63. Epub 2008/12/17. 10.1016/j.vaccine.2008.11.083 19084043 PMC3399430

[60] Guyre PM, Graziano RF, Goldstein J, Wallace PK, Morganelli PM, Wardwell K, et al Increased potency of Fc-receptor-targeted antigens. Cancer Immunol Immunother. 1997;45(3–4):146–8. Epub 1998/01/22. . 9435859 10.1007/s002620050418 PMC11037704

[61] Chen H, Xu X, Jones IM. Immunogenicity of the outer domain of a HIV-1 clade C gp120. Retrovirology. 2007;4:33 Epub 2007/05/19. 10.1186/1742-4690-4-33 17509143 PMC1891314

[62] Schmidt SR. Fusion-proteins as biopharmaceuticals—applications and challenges. Curr Opin Drug Discov Devel. 2009;12(2):284–95. Epub 2009/04/01. . 19333874

[63] Pleass RJ. Fc-receptors and immunity to malaria: from models to vaccines. Parasite Immunol. 2009;31(9):529–38. Epub 2009/08/21. 10.1111/j.1365-3024.2009.01101.x 19691552 PMC3115686

[64] Czajkowsky DM, Hu J, Shao Z, Pleass RJ. Fc-fusion proteins: new developments and future perspectives. EMBO Mol Med. 2012;4(10):1015–28. Epub 2012/07/28. 10.1002/emmm.201201379 ; PubMed Central PMCID: PMCPmc3491832. 22837174 PMC3491832

